# Quantitative Computed Tomography Parameters in Coronavirus Disease 2019 Patients and Prediction of Respiratory Outcomes Using a Decision Tree

**DOI:** 10.3389/fmed.2022.914098

**Published:** 2022-05-20

**Authors:** Jieun Kang, Jiyeon Kang, Woo Jung Seo, So Hee Park, Hyung Koo Kang, Hye Kyeong Park, Je Eun Song, Yee Gyung Kwak, Jeonghyun Chang, Sollip Kim, Ki Hwan Kim, Junseok Park, Won Joo Choe, Sung-Soon Lee, Hyeon-Kyoung Koo

**Affiliations:** ^1^Division of Pulmonary and Critical Care Medicine, Department of Internal Medicine, Ilsan Paik Hospital, Inje University College of Medicine, Goyang, South Korea; ^2^Division of Infectious Diseases, Department of Internal Medicine, Ilsan Paik Hospital, Inje University College of Medicine, Goyang, South Korea; ^3^Department of Laboratory Medicine, Ilsan Paik Hospital, Inje University College of Medicine, Goyang, South Korea; ^4^Department of Radiology, Ilsan Paik Hospital, Inje University College of Medicine, Goyang, South Korea; ^5^Department of Emergency Medicine, Ilsan Paik Hospital, Inje University College of Medicine, Goyang, South Korea; ^6^Department of Anesthesiology and Pain Medicine, Ilsan Paik Hospital, Inje University College of Medicine, Goyang, South Korea

**Keywords:** coronavirus disease 2019, pneumonia, hypoxia, respiratory failure, quantitative CT, decision tree

## Abstract

**Background:**

Chest computed tomography (CT) scans play an important role in the diagnosis of coronavirus disease 2019 (COVID-19). This study aimed to describe the quantitative CT parameters in COVID-19 patients according to disease severity and build decision trees for predicting respiratory outcomes using the quantitative CT parameters.

**Methods:**

Patients hospitalized for COVID-19 were classified based on the level of disease severity: (1) no pneumonia or hypoxia, (2) pneumonia without hypoxia, (3) hypoxia without respiratory failure, and (4) respiratory failure. High attenuation area (HAA) was defined as the quantified percentage of imaged lung volume with attenuation values between −600 and −250 Hounsfield units (HU). Decision tree models were built with clinical variables and initial laboratory values (model 1) and including quantitative CT parameters in addition to them (model 2).

**Results:**

A total of 387 patients were analyzed. The mean age was 57.8 years, and 50.3% were women. HAA increased as the severity of respiratory outcome increased. HAA showed a moderate correlation with lactate dehydrogenases (LDH) and C-reactive protein (CRP). In the decision tree of model 1, the CRP, fibrinogen, LDH, and gene Ct value were chosen as classifiers whereas LDH, HAA, fibrinogen, vaccination status, and neutrophil (%) were chosen in model 2. For predicting respiratory failure, the decision tree built with quantitative CT parameters showed a greater accuracy than the model without CT parameters.

**Conclusions:**

The decision tree could provide higher accuracy for predicting respiratory failure when quantitative CT parameters were considered in addition to clinical characteristics, PCR Ct value, and blood biomarkers.

## Introduction

The coronavirus disease 2019 (COVID-19) pandemic, resulting from severe acute respiratory syndrome coronavirus 2 (SARS-CoV-2) infection, persists as a major health concern worldwide (1). As of March 12, 2022, over 6 million deaths were recorded from COVID-19 (2). The clinical course of COVID-19 varies, ranging from asymptomatic or mild illness to respiratory failure requiring mechanical ventilation, and in the worst case scenarios leading to death (3–5). In a rapidly evolving situation in which the number of infected persons is increasing at a quick pace (6), predicting patient outcomes remains an important issue in light of the distribution of limited medical resources, and in order to provide the best possible care to patients. In this regard, previous studies highlighted several demographic characteristics and laboratory features as prognostic factors in COVID-19, including older age (7), presence of comorbidities (8), obesity (9), C-reactive protein (CRP) (10), and D-dimer levels (11).

Chest computed tomography (CT) scans play an important role in the diagnosis of COVID-19. In particular, they are useful for identifying characteristic features of COVID-19 pneumonia, such as ground glass opacities (GGOs) and/or consolidations predominantly in peripheral areas which may otherwise be difficult to detect on chest radiographs in some cases (12, 13). It is considered the first-line imaging modality, especially in the initial stages of COVID-19 due to its high sensitivity (14). Recent advances in artificial intelligence have enabled the automatic quantification of various parameters obtained from chest CT images (15–17). These advances may be channeled into developing objective imaging biomarkers for predicting COVID-19 outcomes. Using appropriate density threshold ranges, it is possible to differentiate lung parenchyma involved in COVID-19 from normal lung parenchyma (18).

Information on lung density and airway thickness can be easily obtained using quantitative analysis software powered by a fully automated artificial intelligence algorithm, but there remain insufficient data on whether using this information yields any benefits in addition to clinical/laboratory indicators as regards the prediction of patient clinical severity. This study sought to describe the quantitative CT parameters in COVID-19 patients according to disease severity and assess the correlation between CT and blood biomarkers. We also built decision trees for predicting respiratory outcomes in triage patients using demographic, laboratory, and CT parameters, and assessed the role of quantitative CT parameters.

## Materials and Methods

### Study Subjects and Data Collection

This retrospective cohort study was conducted at Ilsan Paik Hospital, South Korea. Patients admitted with COVID-19 confirmed by reverse transcriptase-polymerase chain reaction (RT-PCR) were enrolled. Patients were excluded if they were admitted after an acute stage of the disease. Baseline characteristics, including age, sex, body mass index (BMI), vaccination history, and comorbidities, were obtained from electronic medical records. Cycle threshold (Ct) values of the RdRp/E genes from RT-PCR and laboratory findings were also acquired.

The study patients were classified based on the level of disease severity: (1) no pneumonia or hypoxia, (2) pneumonia without hypoxia, (3) hypoxia without respiratory failure, and (4) respiratory failure. Pneumonia was defined as radiological evidence of pulmonary infiltrates on chest radiography or chest CT. Hypoxia was defined as an oxygen saturation of <94% on room air at sea level. Respiratory failure was defined as the requirement for oxygen supply *via* a high-flow nasal cannula, mechanical ventilation, and/or extracorporeal membrane oxygenation. This study was conducted in accordance with the principles of the Declaration of Helsinki. The Institutional Review Board of Ilsan Paik Hospital, Inje University approved of the study protocol (IRB No: 2022-01-025). The need for informed consent was waived given the retrospective nature of the study.

### Laboratory Test Measurements

The following blood tests were performed for all study patients: complete blood cell count with differentials, liver function test, lactate dehydrogenase (LDH), CRP, procalcitonin, fibrinogen, D-dimer, and ferritin. All routine tests were performed in the central laboratory of our hospital within an hour of blood collection. Tests for SARS-CoV-2 were performed using ExiPrep 48 Dx (Bioneer, Daejeon, South Korea) for nucleic acid extraction and STANDARD M nCoV Real-Time Detection Kit (SD Biosensor, Suwon, South Korea) for RT-PCR targeting the RdRp and E genes of SARS-CoV-2. All the test procedures were carried out according to the manufacturers’ instructions.

### Quantitative Chest Computed Tomography Analyses

Chest CT images were obtained in the supine position with standardized CT screening protocols at a tube voltage of 120 kVP and current of 24 mA, which were applied in the high-pitch spiral mode (Aquilion One from Toshiba). Acquired whole-lung images were analyzed using commercial software (Aview^®^ system; Coreline Soft Inc., Seoul, South Korea), which automatically segmented the lungs and detected the airways. High attenuation area (HAA) was defined as the quantified percentage of imaged lung volume with attenuation values between −600 and −250 Hounsfield units (HU), and low attenuation area (LAA) as <−950 HU (19, 20). HAA corresponds to pneumonia-related alterations such as GGOs and consolidation (18). Airway measurements were performed using the full-width-half-maximum (FWHM) method. Details of the airway measurement algorithm for FWHM have been reported previously (21). AWT-Pi10, a surrogate for airway wall thickness, was derived by plotting the square root of the airway wall area against the internal perimeter of each measured airway to assess the theoretical airway with an internal perimeter of 10 mm using a regression line.

### Statistical Analyses

The subjects’ characteristics are presented as means and standard deviations for continuous variables and as relative frequencies and percentages for categorical variables. Statistical analyses were performed using R software (version 3.6.0). Continuous variables were compared using analysis of variance (ANOVA), and categorical variables were compared using a chi-squared test or Fisher’s exact test. For the correlation matrix, Pearson’s correlation between variables was performed using the cor function in the stats package. Linear regression analysis was performed for HAA levels and laboratory values. A violin plot was drawn using the ggplot2 package. To create a decision tree model, patients were divided into training and testing sets with a 7:3 ratio for cross-validation; models were developed in the training set. Two different types of decision tree models were assessed. Model 1 included demographics, Ct values for gene PCR, and blood biomarkers. Model 2 further included the values of quantitative chest CT analysis in addition to model 1. Model 1 was developed in all patients whereas model 2 was built in patients who underwent chest CT. The tree package was used to draw decision trees. A tree is a non-parametric statistical classification procedure that uses a set of if-then-else logical conditions to assign unknown features to a predefined category. Algorithms for constructing a tree work by choosing a variable at each step that best splits the set of items from top to bottom. The tree creates a partition recursively to lower impurities using the Gini index. Gini index measures the probability of a particular variable being incorrectly classified when it is randomly chosen, and it is calculated by subtracting the sum of squared probabilities of each class from 1. The cut-off values of each node were determined as the points that increase the purity of set using the Gini index. The number of pruning nodes was selected using K-fold cross-validation. The accuracy of the tree model was validated using a testing set.

## Results

### Baseline Characteristics

Between September 1 and December 31, 2021, a total of 389 hospitalizations with a diagnosis of COVID-19 were identified. We excluded two patients who were transferred from other hospitals for post-acute care. The mean age of the 387 patients was 57.8 years, and 194 (50.3%) were women. Of these, 204 (52.7%) were fully vaccinated.

The study patients were categorized into four groups based on respiratory outcomes: no pneumonia or hypoxia (*n* = 186, 48.1%), pneumonia without hypoxia (*n* = 116, 30%), hypoxia without respiratory failure (*n* = 66, 17.1%), and respiratory failure (*n* = 19, 4.9%). Table 1 describes the baseline demographic characteristics and findings of the microbiological and laboratory studies according to the respiratory outcome group. Patients with respiratory failure were significantly older than patients without hypoxia irrespective of pneumonia (mean age, 66.2, 56.3, and 56.2 years in patients with respiratory failure, those with pneumonia but not hypoxia, and those without pneumonia, respectively). Sex and BMI were not significantly different between the four groups. Compared to patients without pneumonia, those with pneumonia, hypoxia, or respiratory failure included significantly more patients who were not vaccinated (33.3, 58.6, 62.1, and 63.2%, respectively). The levels of LDH, aspartate aminotransferase (AST), CRP, fibrinogen, neutrophil (%), and ferritin tended to increase as disease severity increased.

**TABLE 1:** Characteristics of COVID-19 patients stratified by severity.

	Total (*n* = 387)	No pneumonia (*n* = 186)	Pneumonia without hypoxia (*n* = 116)	Hypoxia without respiratory failure (*n* = 66)	Respiratory failure (*n* = 19)	*p*
**Demographics**						
Age	57.8 ± 18.2	56.6 ± 18.7^§^	56.3 ± 18.3^§^	61.5 ± 17.3	66.2 ± 12.1*^†^	0.006
Female sex	194 (50.3)	98 (52.7)	55 (47.4)	31 (47.7)	10 (52.6)	0.767
BMI, kg/m^2^	25.4 ± 4.4	25.3 ± 4.5	25.1 ± 4.6	25.7 ± 4.4	26.5 ± 3.3	0.546
Vaccination	204 (52.7)	124 (66.7)	48 (41.4)	25 (37.9)	7 (36.8)	<0.001
**Microbiologic findings**						
RdRp gene	19.0 ± 5.8	19.0 ± 5.8	18.9 ± 6.1	19.8 ± 5.5	16.7 ± 4.1	0.248
E gene	19.3 ± 5.6	19.2 ± 5.8	19.5 ± 5.6	19.6 ± 5.4	17.2 ± 4.0	0.390
**Laboratory findings**						
LDH, U/L	265.8 ± 111.3	218.0 ± 69.9^†^^‡^^§^	258.5 ± 80.1*^‡^^§^	350.2 ± 112.5*^†^^§^	483.1 ± 169.5*^†^⁣^‡^	<0.001
AST, U/L	33.4 ± 21.9	29.0 ± 16.6^‡^^§^	34.2 ± 26.2^§^	37.1 ± 16.2*	59.9 ± 34.3*^†^	<0.001
ALT, U/L	31.9 ± 28.7	30.2 ± 28.8	31.4 ± 29.5	33.3 ± 26.7	46.6 ± 34.6	0.136
CRP, mg/dL	3.2 ± 5.1	1.1 ± 1.5^†^^‡^^§^	2.5 ± 2.5*^‡^^§^	7.1 ± 5.9*^†^	14.2 ± 11.5*^†^	<0.001
Fibrinogen, mg/dL	459.3 ± 141.2	398.1 ± 108.8^†^^‡^^§^	478.0 ± 121.3*^‡^^§^	559.2 ± 158.9*^†^	618.4 ± 140.9*^†^	<0.001
D-dimer, μg/dL	1.0 ± 2.5	0.8 ± 1.9	0.9 ± 2.5	1.6 ± 3.1	2.1 ± 4.5	0.179
WBC,/μL*1000	5.6 ± 3.8	5.4 ± 4.6	5.1 ± 2.2	6.5 ± 2.9	8.3 ± 4.4	0.792
Neutrophil (%)	62.9 ± 13.6	58.7 ± 11.7^‡^^§^	61.5 ± 12.3^‡^^§^	71.9 ± 12.6*^†^^§^	82.6 ± 8.9*^†^⁣^‡^	<0.001
Procalcitonin, ng/mL	1.6 ± 23.7	0.1 ± 0.7	0.4 ± 1.6	7.7 ± 57.7	3.0 ± 6.2	0.035
Ferritin, ng/mL	392.7 ± 419.7	247.3 ± 232.6^†^^‡^^§^	470.2 ± 493.9*	586.6 ± 429.0*	1018.3 ± 661.0*	<0.001

*Data are presented as mean ± standard deviation or number (%).*

*p < 0.05 vs. no pneumonia.

*^†^p < 0.05 vs. pneumonia without hypoxia.*

*^‡^p < 0.05 vs. hypoxia without respiratory failure.*

*^§^ p < 0.05 vs. respiratory failure.*

### Quantitative Chest Computed Tomography Imaging Parameters

A total of 147 patients (38.0%) underwent chest CT scans. The baseline clinical characteristics are shown in Supplementary Table 1. Quantitative chest CT findings are presented in Table 2. The mean value of whole-lung HAA, which reflects the extent of pneumonic infiltration, was 11.7% in all patients. The mean values of whole lung LAA and AWT-Pi10 were 3.4% and 4.0 mm, respectively. The histograms for HAA and LAA in each lobe are represented in Supplementary Figure 1. When calculated in each lobe, HAA tended to be higher in the lower lobes, probably reflecting higher attenuation in dependent areas (Supplementary Figure 2). The mean HAA was 8.7, 8.1, 16.3, 9.6, and 18.3% in the right upper (RUL), right middle (RML), right lower (RLL), left upper (LUL), and left lower (LLL) lobes, respectively.

**TABLE 2:** Quantitative CT findings according to the level of severity.

	Total (*n* = 147)	No pneumonia (*n* = 37)	Pneumonia without hypoxia (*n* = 54)	Hypoxia without respiratory failure (*n* = 42)	Respiratory failure (*n* = 14)	*p*
**CT findings**						
HAA, whole (%)	11.7 ± 7.1	7.7 ± 3.9^‡^^§^	9.7 ± 5.1^‡^^§^	14.2 ± 6.7*^†^^§^	23.0 ± 7.7*^†^⁣^‡^	<0.001
HAA, Rt	11.2 ± 6.7	7.5 ± 3.9^‡^^§^	9.3 ± 4.7^‡^^§^	13.3 ± 6.6*^†^^§^	21.7 ± 7.0*^†^⁣^‡^	<0.001
HAA, Lt	13.1 ± 8.4	8.3 ± 3.7^‡^^§^	10.8 ± 5.9^‡^^§^	15.9 ± 8.0*^†^^§^	25.4 ± 11.1*^†^⁣^‡^	<0.001
LAA, whole (%)	3.4 ± 3.7	4.3 ± 4.2^§^	2.8 ± 3.1^§^	3.8 ± 4.3^§^	1.3 ± 1.1*^†^⁣^‡^	<0.001
AWT-Pi10, mm	4.0 ± 0.9	4.1 ± 0.5	3.8 ± 1.3	4.2 ± 0.4	4.20 ± 0.3	0.144
Wall area (mean)	69.5 ± 5.5	68.9 ± 4.1	68.9 ± 7.1	70.4 ± 4.8	70.4 ± 4.0	0.449

*Data are presented as mean ± standard deviation.*

*p < 0.05 vs. no pneumonia.

*^†^p < 0.05 vs. pneumonia without hypoxia.*

*^‡^p < 0.05 vs. hypoxia without respiratory failure.*

*^§^ p < 0.05 vs. respiratory failure.*

**FIGURE 1:**
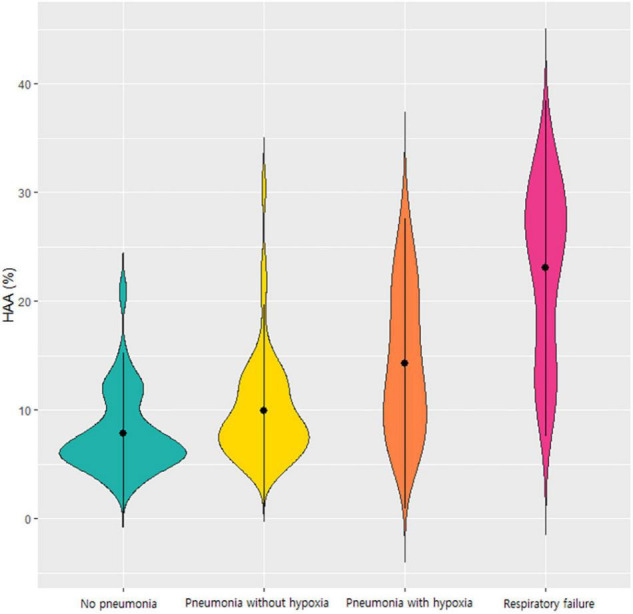
Violin plot of the HAA values according to the respiratory outcomes of COVID-19. Each violin plot represents the distribution of HAA. HAA was sequentially increased in order of patients without pneumonia, those with pneumonia without hypoxia, those with hypoxia without respiratory failure, and those with respiratory failure. Abbreviation: HAA, high attenuation area; COVID-19, Coronavirus disease 2019.

When comparing the groups based on disease severity, HAA increased as the severity of the respiratory outcomes increased. The mean HAA was 7.7% in patients without pneumonia, 9.7% in patients with pneumonia without hypoxia, 14.2% in patients with pneumonia with hypoxia, and 23.0% in patients with respiratory failure. Violin plots representing the distribution of HAA in each group are shown in Figure 1. The HAA level sequentially increased with worsening of respiratory outcomes. The mean LAA was significantly lower in patients with respiratory failure (1.3%) than in the other groups. The mean AWT-Pi10 and wall area did not differ significantly across groups.

### Correlation Between Quantitative Computed Tomography Parameters, Blood Biomarkers, and Respiratory Outcomes

**FIGURE 2:**
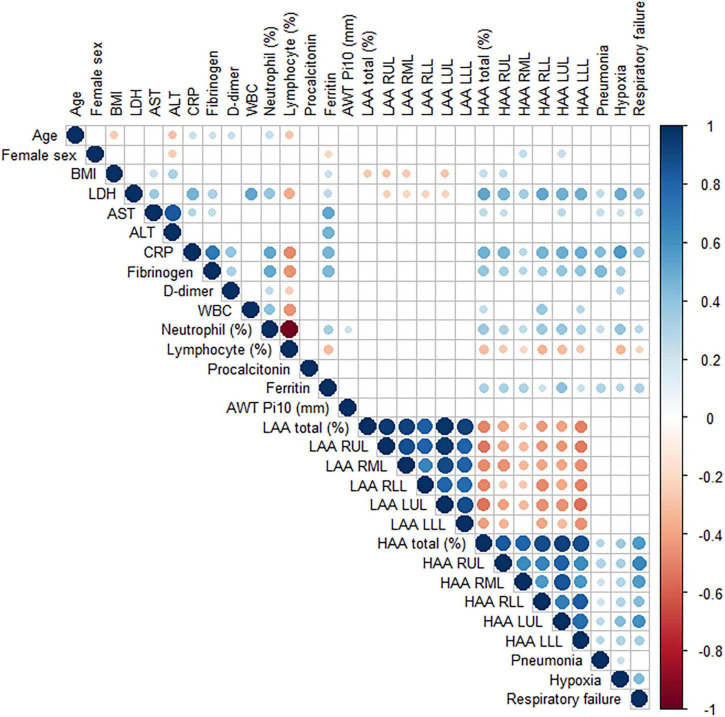
A correlation matrix of demographic, laboratory and quantitative CT image findings. Pearson correlation between variables was performed. The circle sizes and intensity of colors correlate with the strength of their association. The blue and red color indicate positive and negative correlation, respectively. Only statistically significant associations are drawn. Abbreviation: BMI, body mass index; LDH, lactate dehydrogenase; AST, aspartate aminotransferase; ALT, alanine aminotransferase; CRP, C-reactive protein; WBC; white blood cell; LAA, low attenuation area; RUL, right upper lobe; RML, right middle lobe; RLL, right lower lobe; LUL, left upper lobe, LLL; left lower lobe; HAA, high attenuation area.

**FIGURE 3:**
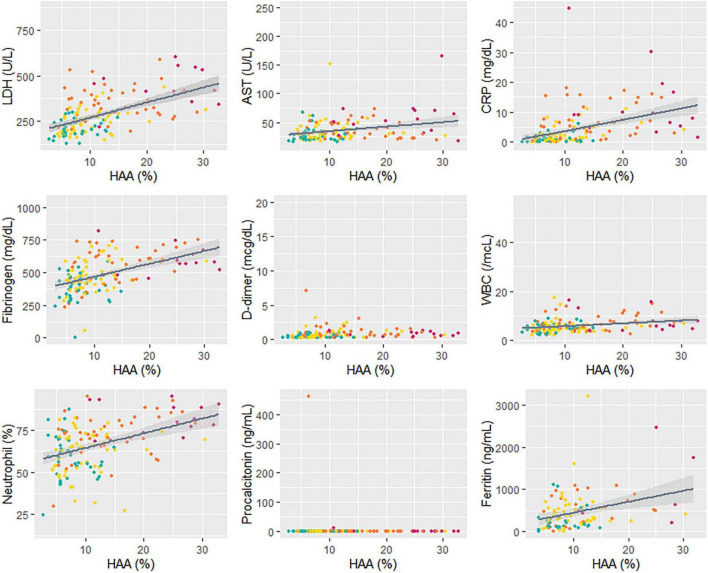
Correlation between HAA and blood biomarkers. A linear regression analysis was performed for total HAA and blood biomarkers. Different colors indicate each respiratory outcome group (green, no pneumonia; yellow, pneumonia without hypoxia; orange, hypoxia without respiratory failure; and red, respiratory failure). The regression coefficients and p-values are as follows: LDH, *R* = 8.84, *p* < 0.001; AST, *R* = 0.76, *p* = 0.002; CRP, *R* = 0.37, *p* < 0.001; fibrinogen, *R* = 9.49, *p* < 0.001; D-dimer, *R* = 0.02, *p* = 0.21; WBC, *R* = 151.1, *p* < 0.001; neutrophil (%), *R* = 0.88, *p* < 0.001; procalcitonin, *R* = 0.004, *p* = 0.75; ferritin, *R* = 24.06, *p* = 0.002. Abbreviation: LDH, lactate dehydrogenase; HAA, high attenuation area; AST, aspartate aminotransferase; CRP, C-reactive protein; WBC; white blood cell.

To understand the association between quantitative CT parameters, blood biomarkers, and clinical features, a correlation matrix was constructed (Figure 2). When associations between respiratory outcomes and quantitative CT or blood biomarkers were assessed, a moderate correlation (a coefficient of 0.4–0.6) was present for pneumonia with CRP and fibrinogen, hypoxia with LDH and CRP, and respiratory failure with HAA (total or each lobe). Total HAA had a moderate correlation (a coefficient of 0.4–0.6) with LDH, CRP, and total LAA, and a weak correlation (a coefficient 0.2–0.4) with AST, fibrinogen, WBC count, neutrophil (%), and ferritin. Detailed values are represented in Supplementary Table 2. Total HAA was chosen as a representative imaging biomarker given its strong correlation with the values of all other lobes (Supplementary Figure 3). Scatter plots showing linear regression analysis of total HAA and blood biomarkers are shown in Figure 3. The LDH, AST, CRP, fibrinogen, WBC count, neutrophil, and ferritin levels were significantly associated with total HAA levels.

### Decision Tree Models to Predict Respiratory Outcomes–Role of the Quantitative Computed Tomography Biomarker

All study patients were randomly allocated to a training and testing set in a 7:3 ratio. A decision tree model for predicting clinical outcomes was developed in the training set and validated in the testing set. Two models were developed: quantitative CT parameters were not considered in model 1 but included in model 2.

**FIGURE 4:**
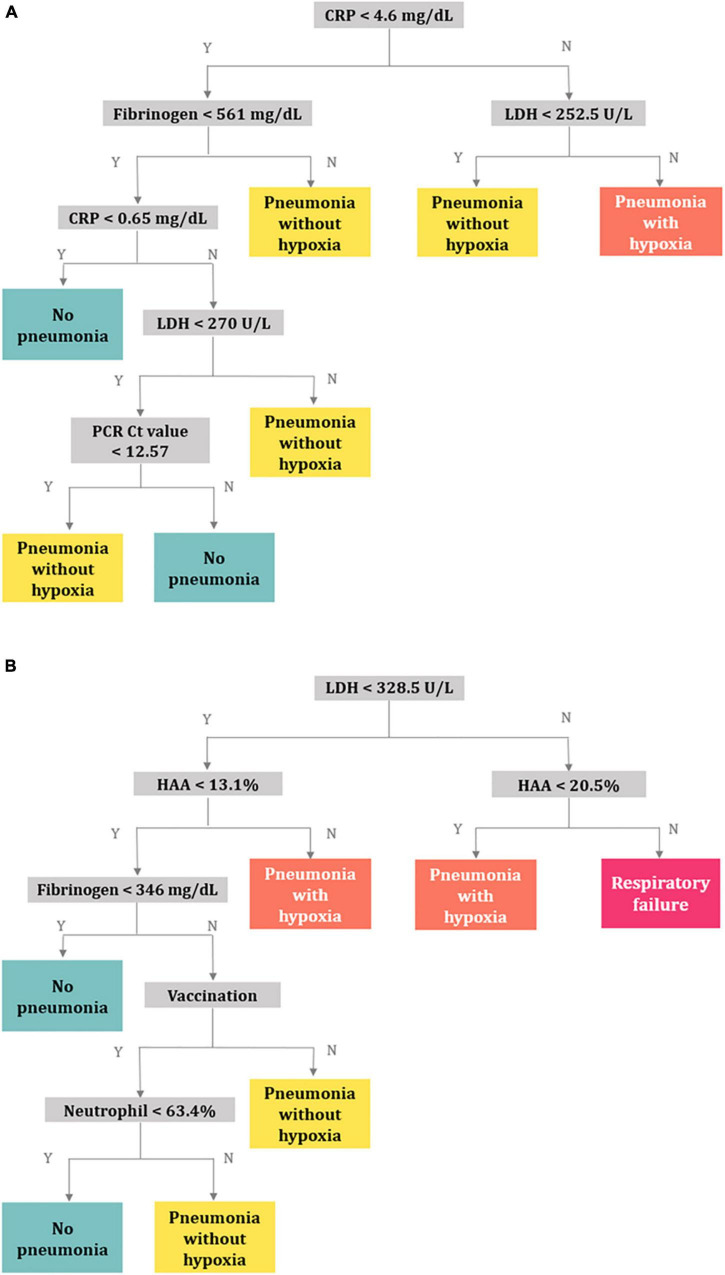
Decision trees for predicting respiratory outcomes. **(A)** Model 1 using the clinical characteristics, PCR Ct values, and laboratory variables, **(B)** model 2 using quantitative CT parameters in addition to the variables in model 1. Abbreviation: PCR, polymerase chain reaction; Ct, cycle threshold; CT, computed tomography; CRP, C-reactive protein; LDH, lactate dehydrogenase; HAA, high-attenuation area.

In model 1, the CRP, fibrinogen, LDH, and Ct values of the RdRp gene were selected as classifiers (Figure 4A). The balanced accuracies for the classification of patients into groups of no pneumonia, pneumonia without hypoxia, hypoxia without respiratory failure, and progression to respiratory failure were 0.739, 0.620, 0.776, and 0.500, respectively. The decision tree of model 1 was not appropriate for predicting respiratory failure given its low accuracy. Further, when the decision tree of model 1 was developed only including patients who underwent chest CT, the balanced accuracies for no pneumonia, pneumonia without hypoxia, hypoxia, and respiratory failure were 0.519, 0.467, 0.681, and 0.500, respectively (Supplementary Figure 4).

In model 2, quantitative CT parameters were included in addition to the variables in model 1, and the following variables were chosen: LDH, HAA, fibrinogen, vaccination status, and neutrophil (%) (Figure 4B). The balanced accuracies for the classification of groups of no pneumonia, pneumonia without hypoxia, pneumonia with hypoxia, and respiratory failure were 0.659, 0.514, 0.671, and 0.807, respectively, which were superior for predicting progression to more severe outcomes than those of model 1. Three-dimensional scatter plots between values of CRP, fibrinogen, and LDH and between values of LDH, HAA, and fibrinogen and respiratory outcomes are represented in Supplementary Figures 5A,B.

## Discussion

This study described the quantitative CT parameters in patients with COVID-19 according to their respiratory outcomes. We also built a simple, easy-to-interpret decision tree to predict respiratory outcomes. The decision tree could provide more accuracy in predicting respiratory failure when quantitative CT parameters are considered in addition to clinical characteristics, PCR Ct values, and blood biomarkers.

The fully automated quantification of CT parameters is being increasingly implemented. In the context of the COVID-19 pandemic, a moderate to strong correlation has been demonstrated between software-driven automatic quantification of the proportion of GGO and/or consolidation and visual assessment of chest CT by radiologists (22). Previous studies have highlighted that quantitative CT biomarkers are useful for predicting COVID-19 outcomes including death (23–27). In a retrospective study including 236 patients with COVID-19, Colombi et al. found that the proportion of well-aerated lung (%) on chest CT predicted adverse outcomes (23). The proportion of well-aerated lung, assessed either visually or by software, was less than 73% on the initial chest CT and was associated with intensive care unit admission or death. Meanwhile, Liu et al. analyzed patients for whom chest CT examinations on days 0 and 4 were available (24). The percentages of GGO volume, semi-consolidation volume, and consolidation volume on chest CT images were obtained using artificial intelligence algorithms in 134 patients diagnosed with COVID-19. They demonstrated that these features on day 0, as well as their changes from day 0 to day 4, could predict the risk of progression to severe illness. In our study, HAA, which represents automatically quantified areas of imaged lung volume with attenuation values between −600 and −250 HU, showed a significant correlation with disease severity and several blood biomarkers and served as a classifier in a decision tree.

In contrast to other studies, the outcomes of COVID-19 infection were divided into four mutually exclusive groups in our study: no pneumonia, pneumonia without hypoxia, hypoxia without respiratory failure, and respiratory failure. This classification may be meaningful for triaging patients in a pandemic situation in which hospital crowding and shortage of beds are of great concern. Indeed, patients without pneumonia or those with no oxygen demand do not necessarily require hospitalization and may be treated at home, whereas patients with hypoxia need to be admitted for supplemental oxygen treatment. In addition, decision trees may provide clues to the pathogenesis of disease progression through their own structure.

Interestingly, a higher HAA level positively corresponded to a greater severity of the disease, supporting its role as a biomarker in predicting COVID-19 severity. In contrast, AWT-Pi10 was not significantly associated with disease severity or other blood biomarkers. One study revealed that patients with COVID-19 have more frequent airway thickening, measured by wall area (%) and AWT-Pi10 compared with those without COVID-19, but there was no significant association between airway thickness and disease severity (28). The authors suggested that this finding likely results from the fact that the primary structures attacked by SARS-CoV-2 are alveoli, not airways (28).

Several blood biomarkers have been linked to disease severity. Pneumonia was found to be correlated with CRP and fibrinogen levels, whereas hypoxia was correlated with LDH and CRP levels. LDH is considered a general marker of cell or tissue injury and reflects the severity of inflammation. Elevated LDH levels are a predictor of severe COVID-19 (29–31). Fibrinogen has also been shown to be higher in patients with severe disease than in those without severe disease (32) and to predict poor prognosis in patients with COVID-19 (33). When the decision tree model was created with relevant variables without quantitative CT parameters, the selected classifiers were CRP, fibrinogen, LDH, and Ct values of the RdRp gene, all of which were considered to represent the severity of inflammation. However, the discriminative power of the decision tree for severe respiratory outcomes was higher in model 2, where quantitative CT parameters were considered in addition to the variables in model 1, supporting the role of quantitative CT in predicting the prognosis of COVID-19, especially respiratory failure.

This study had several limitations that need to be addressed. First, this was a retrospective study that included patients from a single center. Further evaluations with a larger patient population are necessary to confirm our findings. Second, the fact that we carried out quantitative CT analyses using a specific software may have limited the widespread clinical application of our results. However, the role of quantitative CT does not seem to be vendor-specific, given the similar results from other studies that used different software. Third, we did not perform a qualitative assessment of chest CT images. We did not evaluate whether specific CT features of COVID-19 (e.g., distribution, consolidation type, or reverse halo sign) were associated with disease severity or outcomes. In addition, visual assessment was not performed. Although HAA may reflect the extent of viral pneumonia, it cannot distinguish other causes with increased density, such as atelectasis or post-inflammatory fibrosis. Interpretation may need to be supplemented with visual assessment in certain patients. However, fully automated quantification enables an easy, fast, and objective assessment in general.

In conclusion, HAA was associated with respiratory outcomes and was found to be strongly correlated with blood biomarkers in patients with COVID-19. The decision tree provided higher accuracy for predicting respiratory failure when quantitative CT parameters were considered in addition to clinical characteristics, PCR Ct values, and blood biomarkers.

## Data Availability Statement

The data analyzed in this study is subject to the following licenses/restrictions: Data are available upon reasonable request to the corresponding author. Requests to access these datasets should be directed to H-KK, gusrud9@yahoo.co.kr.

## Ethics Statement

The studies involving human participants were reviewed and approved by the Institutional Review Board of Ilsan Paik Hospital, Inje University approved of the study protocol (IRB No: 2022-01-025). Written informed consent for participation was not required for this study in accordance with the national legislation and the institutional requirements.

## Author Contributions

H-KK serves as a guarantor and takes full responsibility for the content of this manuscript and including the data and analysis. H-KK and JK contributed substantially to the concept and design of the study and wrote the first draft of the manuscript. JiyK, WJS, HKK, SHP, HKP, S-SL, JES, and YGK were responsible for the data collection. JC, SK, KHK, SP, and WJC made substantial contribution to the data analysis and interpretation. All authors discussed the results and reviewed the manuscript.

## Conflict of Interest

The authors declare that the research was conducted in the absence of any commercial or financial relationships that could be construed as a potential conflict of interest.

## Publisher’s Note

All claims expressed in this article are solely those of the authors and do not necessarily represent those of their affiliated organizations, or those of the publisher, the editors and the reviewers. Any product that may be evaluated in this article, or claim that may be made by its manufacturer, is not guaranteed or endorsed by the publisher.

## References

[1] Pollard CA Morran MP Nestor-Kalinoski AL. The COVID-19 pandemic: a global health crisis. *Physiol Genomics.* (2020) 52:549–57. 32991251 10.1152/physiolgenomics.00089.2020 PMC7686876

[2] World Health Organization. *WHO Coronavirus (COVID-19) Dashboard.* Geneva: World Health Organization (2022).

[3] García LF. Immune response, inflammation, and the clinical spectrum of COVID-19. *Front Immunol.* (2020) 11:1441. 10.3389/fimmu.2020.01441 32612615 PMC7308593

[4] Eythorsson E Helgason D Ingvarsson RF Bjornsson HK Olafsdottir LB Bjarnadottir V Clinical spectrum of coronavirus disease 2019 in Iceland: population based cohort study. *BMJ.* (2020) 371:m4529. 10.1136/bmj.m452 33268329 PMC7708618

[5] Chen N Zhou M Dong X Qu J Gong F Han Y Epidemiological and clinical characteristics of 99 cases of 2019 novel coronavirus pneumonia in Wuhan, China: a descriptive study. *Lancet.* (2020) 395:507–13. 10.1016/S0140-6736(20)30211-7 32007143 PMC7135076

[6] Viana R Moyo S Amoako DG Tegally H Scheepers C Althaus CL Rapid epidemic expansion of the SARS-CoV-2 Omicron variant in southern Africa. *Nature.* (2022) 603:679–86. 10.1038/s41586-022-04411-y 35042229 PMC8942855

[7] Izcovich A Ragusa MA Tortosa F Lavena Marzio MA Agnoletti C Bengolea A Prognostic factors for severity and mortality in patients infected with COVID-19: a systematic review. *PLoS One.* (2020) 15:e0241955. 10.1371/journal.pone.0241955 33201896 PMC7671522

[8] Fathi M Vakili K Sayehmiri F Mohamadkhani A Hajiesmaeili M Rezaei-Tavirani M The prognostic value of comorbidity for the severity of COVID-19: a systematic review and meta-analysis study. *PLoS One.* (2021) 16:e0246190. 10.1371/journal.pone.0246190 33592019 PMC7886178

[9] Tamara A Tahapary DL. Obesity as a predictor for a poor prognosis of COVID-19: a systematic review. *Diabetes Metab Syndr.* (2020) 14:655–9. 10.1016/j.dsx.2020.05.020 32438328 PMC7217103

[10] Stringer D Braude P Myint PK Evans L Collins JT Verduri A The role of C-reactive protein as a prognostic marker in COVID-19. *Int J Epidemiol.* (2021) 50:420–9. 33683344 10.1093/ije/dyab012 PMC7989395

[11] Rostami M Mansouritorghabeh H. D-dimer level in COVID-19 infection: a systematic review. *Expert Rev Hematol.* (2020) 13:1265–75. 10.1080/17474086.2020.1831383 32997543

[12] Chung M Bernheim A Mei X Zhang N Huang M Zeng X CT imaging features of 2019 novel coronavirus (2019-nCoV). *Radiology.* (2020) 295:202–7. 10.1148/radiol.2020200230 32017661 PMC7194022

[13] Li M Lei P Zeng B Li Z Yu P Fan B Coronavirus disease (COVID-19): spectrum of CT findings and temporal progression of the disease. *Acad Radiol.* (2020) 27:603–8. 10.1016/j.acra.2020.03.003 32204987 PMC7156150

[14] Li Y Xia L. Coronavirus disease 2019 (COVID-19): role of chest CT in diagnosis and management. *AJR Am J Roentgenol.* (2020) 214:1280–6. 10.2214/AJR.20.22954 32130038

[15] Obert M Kampschulte M Limburg R Barańczuk S Krombach GA. Quantitative computed tomography applied to interstitial lung diseases. *Eur J Radiol.* (2018) 100:99–107. 10.1016/j.ejrad.2018.01.018 29496086

[16] Humphries SM Notary AM Centeno JP Strand MJ Crapo JD Silverman EK Deep learning enables automatic classification of emphysema pattern at CT. *Radiology.* (2020) 294:434–44. 10.1148/radiol.2019191022 31793851 PMC6996603

[17] Lynch DA Al-Qaisi MA. Quantitative computed tomography in chronic obstructive pulmonary disease. *J Thorac Imaging.* (2013) 28:284–90. 10.1097/rti.0b013e318298733c 23748651 PMC4161463

[18] Annoni AD Conte E Mancini ME Gigante C Agalbato C Formenti A Quantitative evaluation of COVID-19 pneumonia lung extension by specific software and correlation with patient clinical outcome. *Diagnostics (Basel).* (2021) 11:265. 10.3390/diagnostics11020265 33572122 PMC7915160

[19] Lederer DJ Enright PL Kawut SM Hoffman EA Hunninghake G van Beek EJ Cigarette smoking is associated with subclinical parenchymal lung disease: the multi-ethnic study of atherosclerosis (MESA)-lung study. *Am J Respir Crit Care Med.* (2009) 180:407–14. 10.1164/rccm.200812-1966OC 19542480 PMC2742759

[20] Romanov A Bach M Yang S Franzeck FC Sommer G Anastasopoulos C Automated CT lung density analysis of viral pneumonia and healthy lungs using deep learning-based segmentation, histograms and HU thresholds. *Diagnostics (Basel).* (2021) 11:738. 10.3390/diagnostics11050738 33919094 PMC8143124

[21] Park HJ Lee SM Choe J Lee SM Kim N Lee JS Prediction of treatment response in patients with chronic obstructive pulmonary disease by determination of airway dimensions with baseline computed tomography. *Korean J Radiol.* (2019) 20:304–12. 10.3348/kjr.2018.0204 30672170 PMC6342755

[22] Shen C Yu N Cai S Zhou J Sheng J Liu K Quantitative computed tomography analysis for stratifying the severity of coronavirus disease 2019. *J Pharm Anal.* (2020) 10:123–9. 10.1016/j.jpha.2020.03.004 32292624 PMC7102584

[23] Colombi D Bodini FC Petrini M Maffi G Morelli N Milanese G Well-aerated lung on admitting chest ct to predict adverse outcome in COVID-19 pneumonia. *Radiology.* (2020) 296:E86–96. 10.1148/radiol.2020201433 32301647 PMC7233411

[24] Liu F Zhang Q Huang C Shi C Wang L Shi N CT quantification of pneumonia lesions in early days predicts progression to severe illness in a cohort of COVID-19 patients. *Theranostics.* (2020) 10:5613–22. 10.7150/thno.45985 32373235 PMC7196293

[25] Morris MF Pershad Y Kang P Ridenour L Lavon B Lanclus M Altered pulmonary blood volume distribution as a biomarker for predicting outcomes in COVID-19 disease. *Eur Respir J.* (2021) 58:2004133. 10.1183/13993003.04133-2020 33632795 PMC7908189

[26] Sun D Li X Guo D Wu L Chen T Fang Z CT quantitative analysis and its relationship with clinical features for assessing the severity of patients with COVID-19. *Korean J Radiol.* (2020) 21:859–68. 10.3348/kjr.2020.0293 32524786 PMC7289692

[27] Lanza E Muglia R Bolengo I Santonocito OG Lisi C Angelotti G Quantitative chest CT analysis in COVID-19 to predict the need for oxygenation support and intubation. *Eur Radiol.* (2020) 30:6770–8. 10.1007/s00330-020-07013-2 32591888 PMC7317888

[28] Xu J Liang Z Jian W Li J Tang G Mo X Changes of quantitative CT-based airway wall dimensions in patients with COVID-19 during early recovery. *J Thorac Dis.* (2021) 13:1517–30. 10.21037/jtd-20-2790 33841944 PMC8024853

[29] Yang Q Li J Zhang Z Wu X Liao T Yu S Clinical characteristics and a decision tree model to predict death outcome in severe COVID-19 patients. *BMC Infect Dis.* (2021) 21:783. 10.1186/s12879-021-06478-w 34372767 PMC8351764

[30] Ke C Yu C Yue D Zeng X Hu Z Yang C. Clinical characteristics of confirmed and clinically diagnosed patients with 2019 novel coronavirus pneumonia: a single-center, retrospective, case-control study. *Med Clin (Barc).* (2020) 155:327–34. 10.1016/j.medcli.2020.06.055 32782109 PMC7386390

[31] Wang L Yang LM Pei SF Chong YZ Guo Y Gao XL CRP, SAA, LDH, and DD predict poor prognosis of coronavirus disease (COVID-19): a meta-analysis from 7739 patients. *Scand J Clin Lab Invest.* (2021) 81:679–86. 10.1080/00365513.2021.2000635 34762008

[32] Gao Y Li T Han M Li X Wu D Xu Y Diagnostic utility of clinical laboratory data determinations for patients with the severe COVID-19. *J Med Virol.* (2020) 92:791–6. 10.1002/jmv.25770 32181911 PMC7228247

[33] Sui J Noubouossie DF Gandotra S Cao L. Elevated plasma fibrinogen is associated with excessive inflammation and disease severity in COVID-19 patients. *Front Cell Infect Microbiol.* (2021) 11:734005. 10.3389/fcimb.2021.734005 34414135 PMC8369350

