# Supplementary material for: Quantitative Computed Tomography Parameters in Coronavirus Disease 2019 Patients and Prediction of Respiratory Outcomes Using a Decision Tree
Source: Front Med (Lausanne). 2022 May 20;9:914098. doi: 10.3389/fmed.2022.914098 (PMC9163736; doi:10.3389/fmed.2022.914098)
Supplement: Supplementary file 1 [file Data_Sheet_1.docx]

**Supplementary File**

**Appendix to: Quantitative Computed Tomography Parameters in Coronavirus Disease 2019 Patients and Prediction of Respiratory Outcomes using Decision Tree**

Supplementary Table S1. Characteristics of COVID-19 patients in whom chest computed tomography scan was performed

|  | n = 147 |
| --- | --- |
| **Demographics** |  |
| Age | 60.0 ± 17.7 |
| Female sex | 72 (49.0%) |
| Body mass index, kg/m^2^ | 25.7 ± 4.6 |
| Vaccination | 67 (45.6%) |
| **Microbiologic findings: Ct values** | |
| RdRp gene | 18.9 ± 5.6 |
| E gene | 18.9 ± 5.4 |
| **Laboratory findings** | |
| LDH, U/L | 290.3 ± 114.8 |
| AST, U/L | 35.9 ± 20.6 |
| ALT, U/L | 32.8 ± 29.6 |
| CRP, mg/dL | 4.4 ± 6.1 |
| Fibrinogen, mg/dL | 482.3 ± 142.2 |
| D-dimer, μg/dL | 0.8 ± 0.8 |
| WBC, /μL*1000 | 6.1 ± 3.8 |
| Neutrophil (%) | 65.9 ± 15.0 |
| Procalcitonin, ng/mL | 3.5 ± 38.6 |
| Ferritin, ng/mL | 464.3 ± 481.3 |

Data are presented as mean ± standard deviation or number (%).

Abbreviation: COVID-19, coronavirus 2019; Ct, cycle threshold; LDH, lactate dehydrogenase; AST, aspartate aminotransferase; ALT, alanine aminotransferase; CRP, C-reactive protein; WBC; white blood cell.

Supplementary Table S2. Correlation matrix between variables

|  | LDH | AST | ALT | CRP | Fibrinogen | D-dimer | WBC | Neutrophil | Ferritin | HAA total | Pneumonia | Hypoxia | Resp failure |
| --- | --- | --- | --- | --- | --- | --- | --- | --- | --- | --- | --- | --- | --- |
| LDH | 1 | 0.363 | 0.092 | 0.457 | 0.297 | 0.193 | 0.505 | 0.400 | 0.257 | 0.504 | 0.228 | 0.491 | 0.376 |
| AST |  | 1 | 0.846 | 0.293 | 0.245 | 0.156 | -0.020 | 0.194 | 0.508 | 0.244 | 0.249 | 0.220 | 0.251 |
| ALT |  |  | 1 | 0.054 | 0.075 | 0.062 | 0.041 | 0.115 | 0.474 | 0.088 | 0.139 | 0.034 | 0.061 |
| CRP |  |  |  | 1 | 0.696 | 0.379 | 0.161 | 0.521 | 0.463 | 0.466 | 0.425 | 0.556 | 0.368 |
| Fibrinogen |  |  |  |  | 1 | 0.307 | 0.057 | 0.510 | 0.459 | 0.374 | 0.439 | 0.326 | 0.170 |
| D-dimer |  |  |  |  |  | 1 | 0.180 | 0.241 | 0.183 | 0.069 | 0.182 | 0.263 | -0.023 |
| WBC |  |  |  |  |  |  | 1 | 0.430 | 0.033 | 0.224 | -0.086 | 0.108 | -0.040 |
| Neutrophil (%) | |  |  |  |  |  |  | 1 | 0.336 | 0.331 | 0.243 | 0.386 | 0.277 |
| Ferritin |  |  |  |  |  |  |  |  | 1 | 0.312 | 0.331 | 0.288 | 0.307 |
| HAA total (%) | |  |  |  |  |  |  |  |  | 1 | 0.296 | 0.339 | 0.579 |
| Pneumonia | |  |  |  |  |  |  |  |  |  | 1 | 0.215 | 0.184 |
| Hypoxia |  |  |  |  |  |  |  |  |  |  |  | 1 | 0.431 |
| Respiratory failure | |  |  |  |  |  |  |  |  |  |  |  | 1 |

Pearson correlation coefficient values are presented.

Abbreviation: LDH, lactate dehydrogenase; AST, aspartate aminotransferase; ALT, alanine aminotransferase; CRP, C-reactive protein; WBC; white blood cell; HAA, high attenuation area; Resp, respiratory.

Supplementary Figure S1. Histogram of proportion of HAA and LAA according to severity of COVID-19 pneumonia


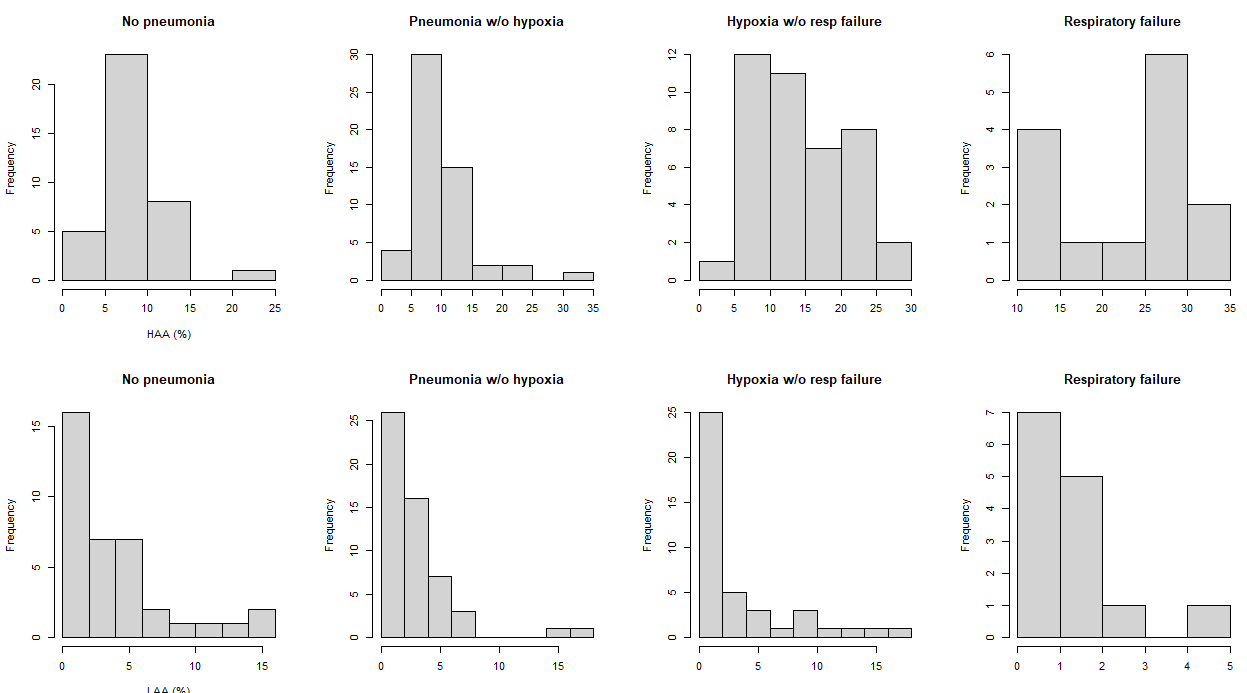


Abbreviation: HAA, high-attenuation area between -600 and -250 HU; LAA, low-attenuation area lower than -950 HU; COVID-19, coronavirus disease 2019.

Supplementary Figure S2. Distribution of HAA values of each lobe comparing each disease severity


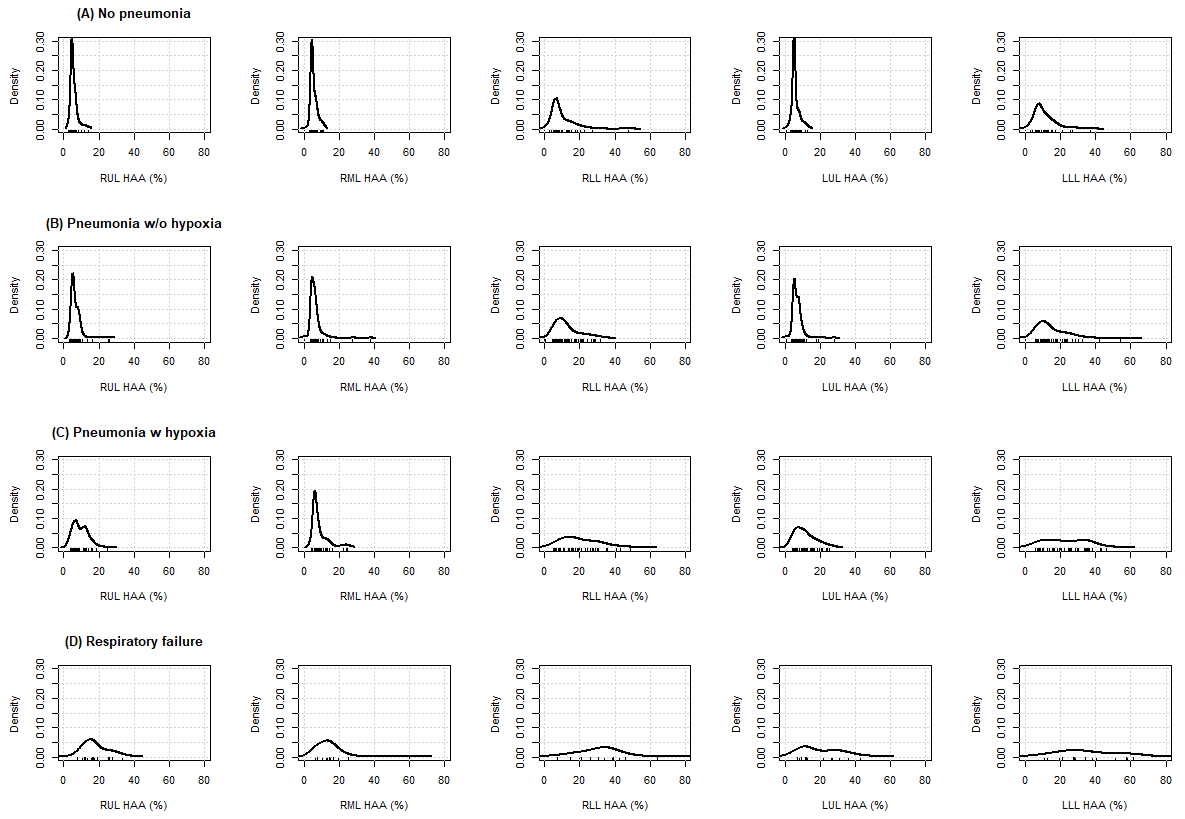


Abbreviation: HAA, high-attenuation area between -600 and -250 HU; LAA, low-attenuation area lower than -950 HU; COVID-19, coronavirus disease 2019.

Supplementary Figure S3. Correlation between quantitative CT values of HAA


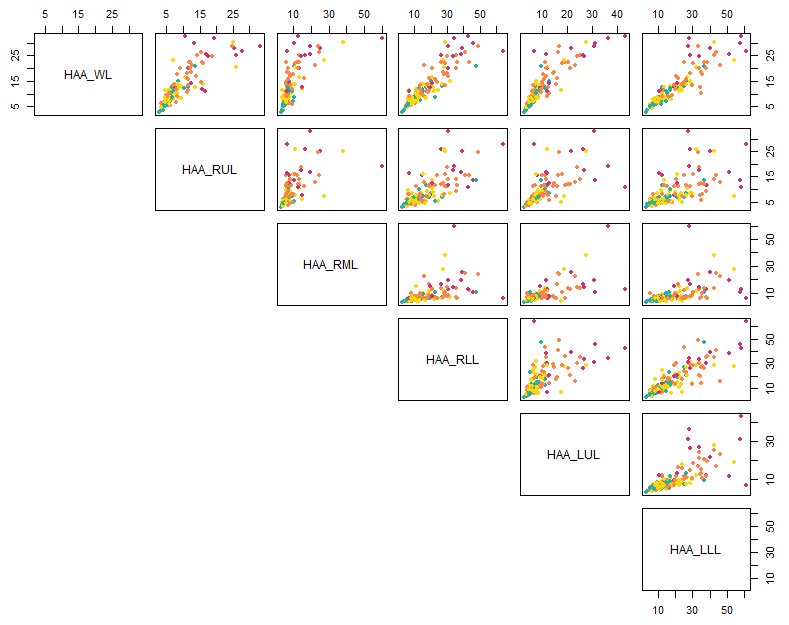


Abbreviation: CT, computed tomography; HAA, high attenuation area.

Different color indicates each group (green, no pneumonia; yellow, pneumonia without hypoxia; orange, hypoxia without respiratory failure; and red, respiratory failure).

Supplementary Figure S4. Decision trees of model 1 in patients who underwent chest CT scan


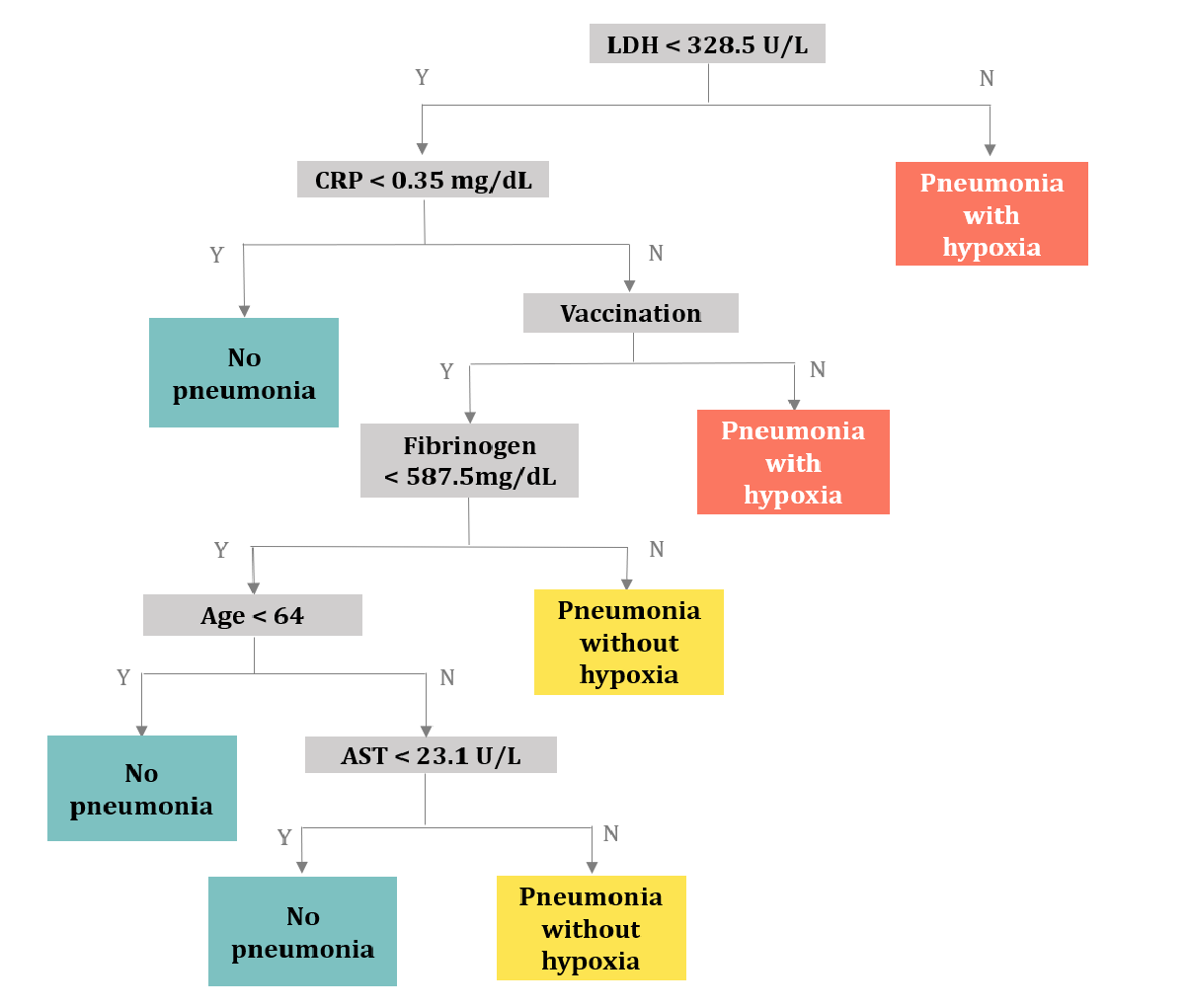


Supplementary Figure S5. Association between values of (A) CRP, fibrinogen, and LDH, and (B) LDH, HAA (%), and fibrinogen with respiratory outcomes

(A) X, Y, and Z axis mean CRP, fibrinogen, and LDH, respectively.


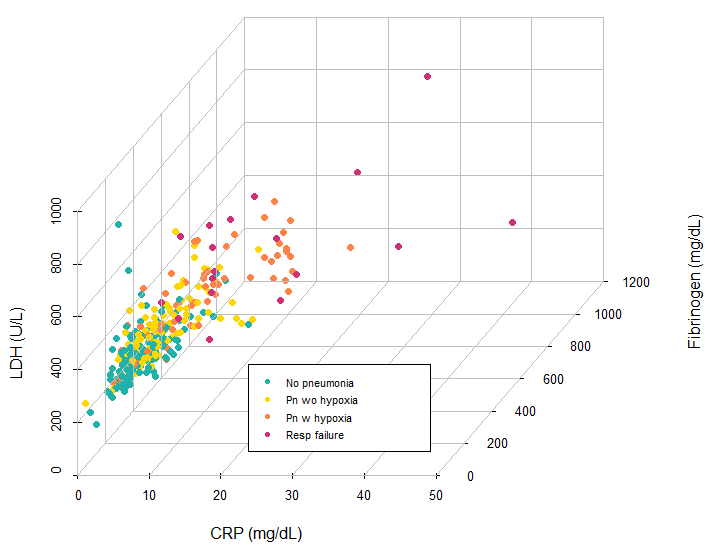


(B) X, Y, and Z axis mean LDH, HAA, and fibrinogen, respectively.


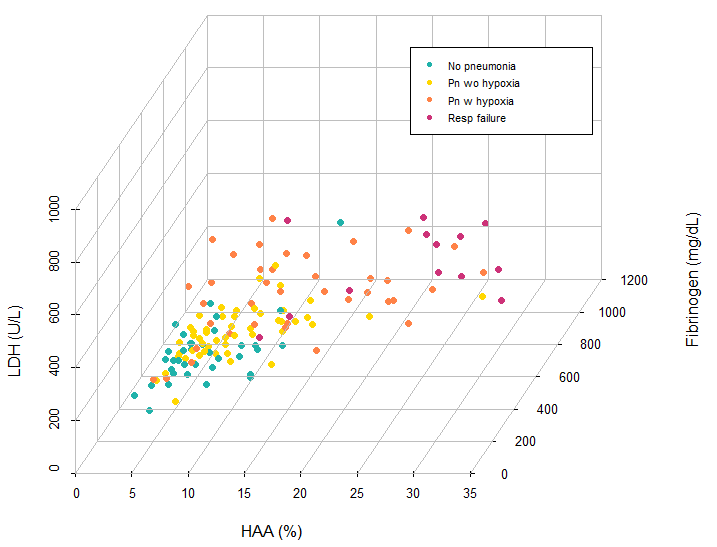


Different color indicates each group (green, no pneumonia; yellow, pneumonia without hypoxia; orange, hypoxia without respiratory failure; and red, respiratory failure).

Abbreviation: CRP, C-reactive protein; LDH, lactate dehydrogenase; HAA, high attenuation area; Pn, pneumonia; wo, without; w, with; resp, respiratory.
